# MDcons: Intermolecular contact maps as a tool to analyze the interface of protein complexes from molecular dynamics trajectories

**DOI:** 10.1186/1471-2105-15-S5-S1

**Published:** 2014-05-06

**Authors:** Safwat Abdel-Azeim, Edrisse Chermak, Anna Vangone, Romina Oliva, Luigi Cavallo

**Affiliations:** 1 Kaust Catalysis Center, King Abdullah University of Science and Technology, Thuwal 23955-6900, Saudi Arabia; 2 Department of Chemistry and Biology, University of Salerno, Via Ponte don Melillo 84084, Fisciano (SA), Italy; 3 Department of Sciences and Technologies, University "Parthenope" of Naples, Centro Direzionale Isola C4 80143, Naples, Italy

## Abstract

**Background:**

Molecular Dynamics (MD) simulations of protein complexes suffer from the lack of specific tools in the analysis step. Analyses of MD trajectories of protein complexes indeed generally rely on classical measures, such as the RMSD, RMSF and gyration radius, conceived and developed for single macromolecules. As a matter of fact, instead, researchers engaged in simulating the dynamics of a protein complex are mainly interested in characterizing the conservation/variation of its biological interface.

**Results:**

On these bases, herein we propose a novel approach to the analysis of MD trajectories or other conformational ensembles of protein complexes, MDcons, which uses the conservation of inter-residue contacts at the interface as a measure of the similarity between different snapshots. A "consensus contact map" is also provided, where the conservation of the different contacts is drawn in a grey scale. Finally, the interface area of the complex is monitored during the simulations. To show its utility, we used this novel approach to study two protein-protein complexes with interfaces of comparable size and both dominated by hydrophilic interactions, but having binding affinities at the extremes of the experimental range. MDcons is demonstrated to be extremely useful to analyse the MD trajectories of the investigated complexes, adding important insight into the dynamic behavior of their biological interface.

**Conclusions:**

MDcons specifically allows the user to highlight and characterize the dynamics of the interface in protein complexes and can thus be used as a complementary tool for the analysis of MD simulations of both experimental and predicted structures of protein complexes.

## Background

The thousands of proteins expressed in cells perform many of their functions through interactions with other proteins. Understanding protein-protein interactions is a crucial step in the investigation of many processes, such as intracellular signaling pathways [1], antibody-antigen pairing [2], enzyme-inhibitor interactions [3]. Therefore, the detailed characterization on a structural basis of protein-protein complexes has become an important task for both wet and computational biologists. Thousands of experimental structures of protein-protein complexes are currently available from the wwPDB [4] and many more can be reliably predicted by computational approaches, as shown in the Critical Assessment of PRedicted Interactions (CAPRI) experiment [5,6].

The dynamical characterization of such complexes can add valuable information on the recognition process, and Molecular dynamics (MD) has indeed been long established as a useful tool to help understanding biological process at the atomic and molecular levels [7-10]. The recent advances in the computational power and the development of new theoretical methods have moreover made MD simulations of large systems on a large time scale much more affordable than before [11-13]. However, MD simulations of protein complexes suffer from the lack of specific tools in the analysis step. The analysis of MD trajectories of protein complexes indeed generally relies on classical measures, such as root-mean-square deviation (RMSD), root-mean-square-fluctuation (RMSF) and radius of gyration, to list a few, conceived and developed for single macromolecules. As a matter of fact, instead, researchers engaged in simulating the dynamics of a protein complex are mainly interested in characterizing the conservation/variation of its biological interface.

Whereas a large number of specific tools have been developed to analyze single biomolecules, for example to measure the volume of pockets in proteins (Mdpocket [14] and POVME [15]), to analyze their hydrogen bonds network (HBonanza [16]) or to characterize the fine structural details of DNA molecules (MDDNA[17] ) during MD simulations, there is a paucity of methods to specifically analyse MD trajectories of protein complexes. In this context, we have previously shown that intermolecular contact maps (i.e. maps where a black dot is present at the cross-over of two residues belonging to the two interacting molecules if any pair of atoms belonging to the two residues is closer than a chosen cut-off distance), can be successful applied to the analysis of both experimental and predicted structures of protein-protein complexes [18-21]. In particular, we have shown that an intermolecular contact map can identify uniquely and intuitively the surface of interaction, representing a sort of fingerprint of the complex and reporting the crucial information in a ready-to-read form. It therefore allows to easily and intuitively discriminate between similar and different binding solutions [18]. Furthermore, we have shown that intermolecular contact maps, together with the measure of the conservation of inter-molecular contacts, can be used to analyse docking model ensembles [19] and to reliably extract from them the native-like solutions [20,21].

Herein we extend this approach to the analysis of MD trajectories of protein complexes. We use the conservation of inter-residue contacts (ICs) at the interface as a measure of the similarity between different snapshots. A "consensus contact map" is also provided, where the conservation of the different contacts is drawn in a grey scale, and the complex interface area along the simulation is monitored. This novel approach is embodied in an algorithm we have named MDcons (Molecular Dynamics CONSensus). In the following, as exemplary cases, we apply MDcons to study two protein-protein complexes with comparable interface areas and binding affinities at the extremes of the experimental range. We show that differences in the dynamic behavior of the analyzed systems, particularly at their biological interface, are efficiently outlined by the MDcons approach. As a final remark, we note that the importance of inter-residue contacts when analyzing 3D structures of protein complexes is well established. In the CAPRI experiment, for instance, the correctness of a prediction, i.e. its similarity to the native structure, is assessed based on a combination of RMSD criteria and of conservation of inter-residue contacts, as compared to the native structure [5,22]. Furthermore, the fraction of common inter-residue contacts among a set of docking models has been recently shown to successfully apply to their clustering [23].

## Results and discussion

We developed a novel tool, MDcons, specifically devoted to the analysis of MD trajectories for protein complexes, which is based on the conservation of ICs during a MD simulation and on its visualization in the form of an intermolecular contact map, that we made available at https://www.molnac.unisa.it/BioTools/mdcons/index.php/MDcons. We have applied this novel approach to two protein-protein complexes sharing interfaces of similar size, about 700 Å^2^, and both dominated by hydrophilic interactions, while dramatically differing in their experimentally measured binding affinities (by over 10 kcal/mol, see Table 1[24]). The high binding affinity system corresponds to the ColE7- Im2 complex (PDB code: 7CEI [25]), while the low binding affinity one corresponds to the CD2-CD48 complex (PDB code: 1QA9 [26]). Their binding affinities are -19.50 and -7.16 kcal/mol, respectively, being at the extremes of the experimental range explored to date, that is approximately -20 to -5 kcal/mol [24].

**Table 1:** Main features of the analysed complexes

PDB ID	Receptor	Ligand	Δ **G (kcal/mol)**	C_50_	C_70_	C_90_	# ICs CR_kl _= 1	MD Interface area (Å^2^)
7CEI	Colicin E7 nuclease	Im7 immunity protein	-19.50	1.0	0.84	0.69	19	733 ± 38
1QA9	CD2	CD58	-7.16	0.95	0.78	0.49	9	641 ± 41

PDB IDs, binding free energies in kcal/mol and MDcons results for the Im7- ColE7 and CD2-CD58 complexes

**Figure 1:**
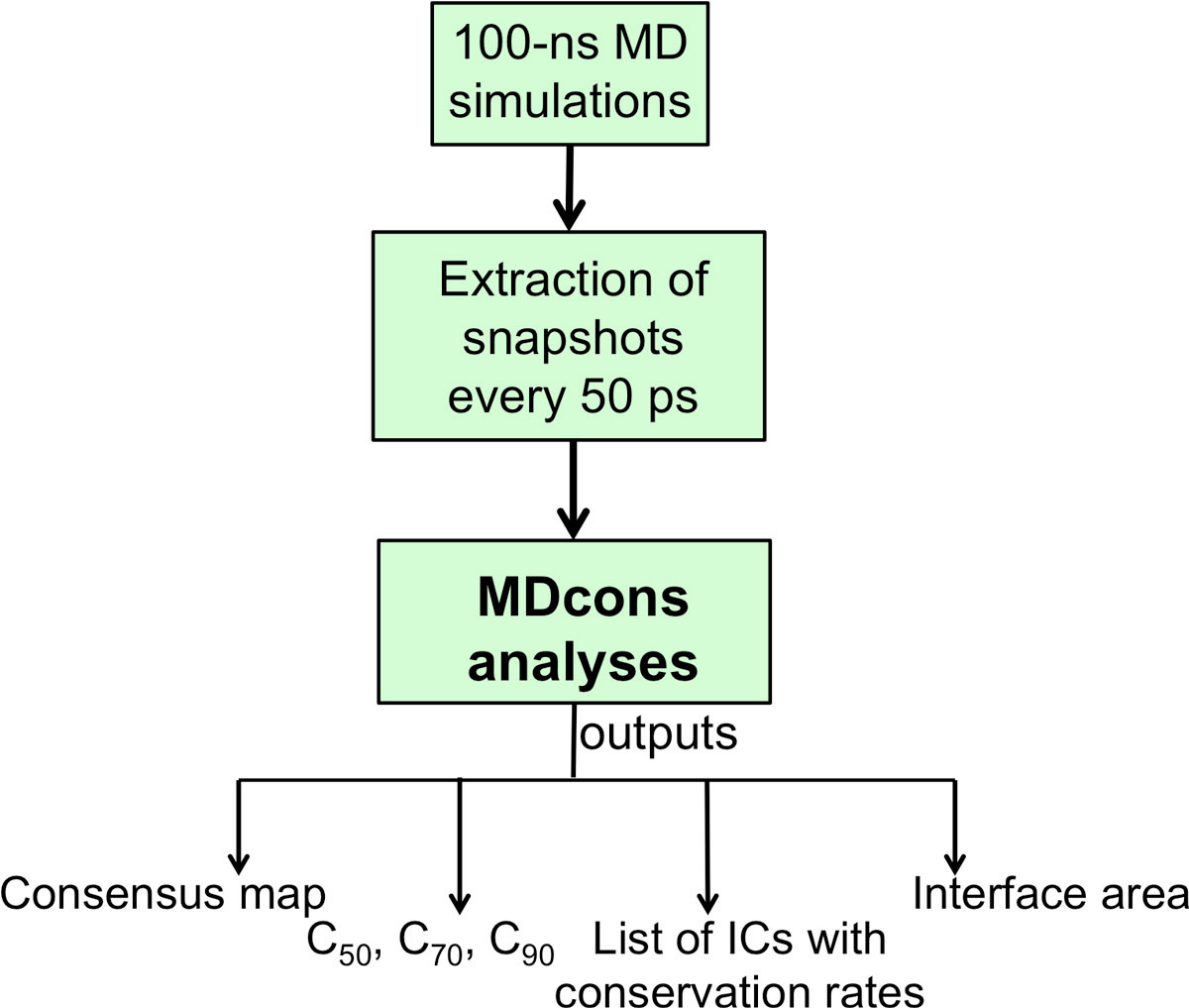
**Schematic representation of the method workflow**.

A scheme of the method workflow is reported in Figure 1. 100 ns-long MD simulations were performed on both complexes as detailed in the Methods section and classical analyses were performed on the obtained trajectories. Then, 2000 snapshots for each structure were extracted every 50 ps and analysed by MDcons. MDcons provides as output: i) a "consensus map", i.e. a 2D map were ICs are shown in a scale of grays were the more conserved the contact, the darker the spot, ii) a list of the ICs with the corresponding conservation rates, CR_kl_, iii) the fraction of ICs which are conserved in at least 50%, 70% and 90% of the analysed snapshots (C_50_, C_70 _and C_90_), representing a measure of the overall conservation of the contacts during the simulations, and iv) the interface area of the complex along the simulation.

### Colicin E7 nuclease - Im7 immunity protein complex (7CEI)

**Figure 2:**
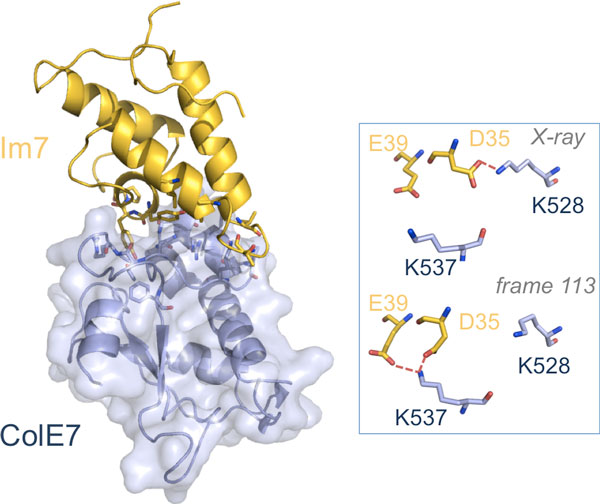
**A 3D representation of the Im7-ColE7 complex (PDB ID: 7CEI)**. Residues involved in ICs with CR_kl _= 1 are shown in a stick representation. *Inset*: the H- bonds network around Asp35/Im7 in the X-ray structure (top) and in frame 113 (bottom) is shown.

**Figure 3:**
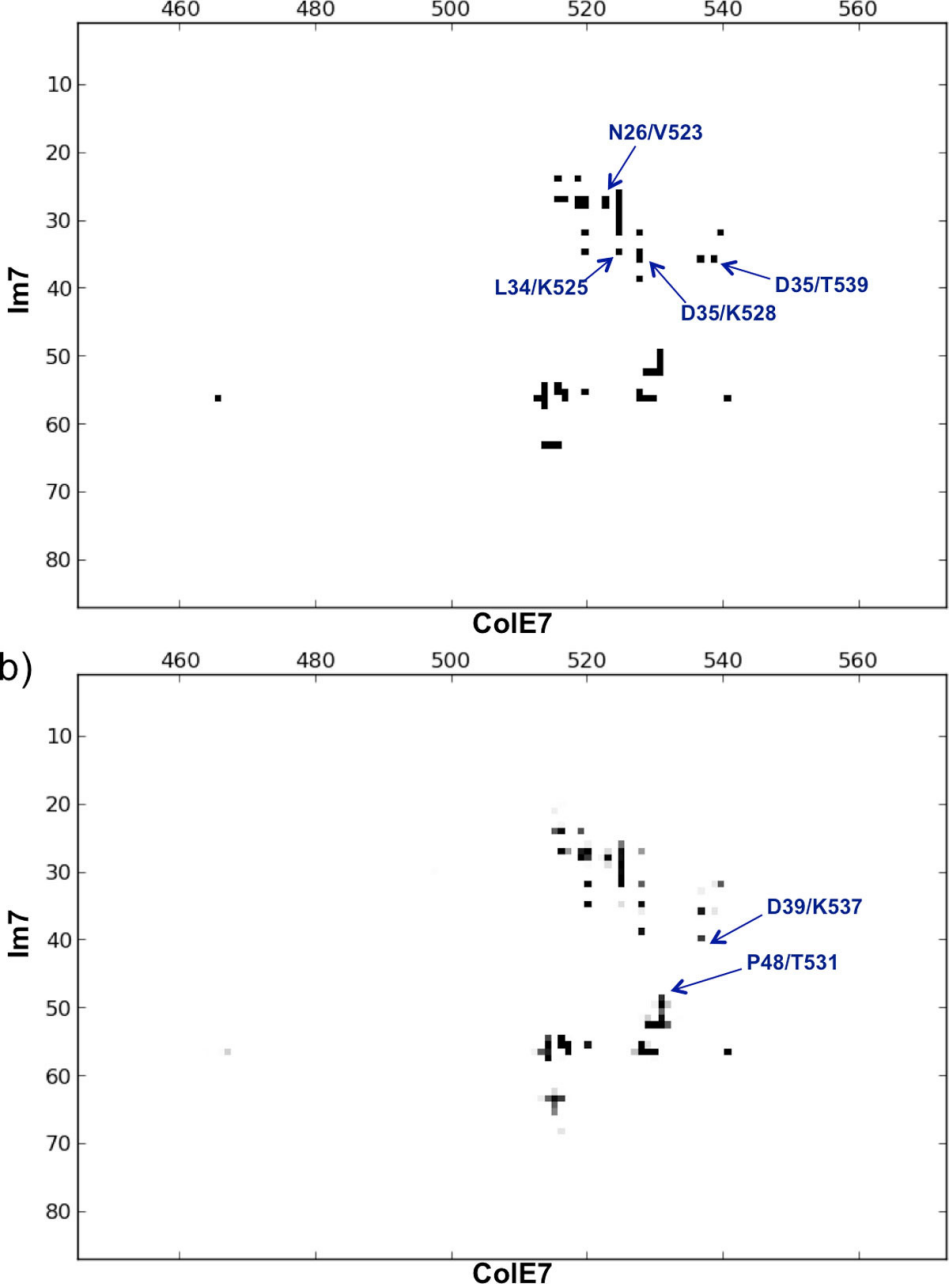
**Comparison between the MDcons consensus map and the intermolecular contact map of the Im7-ColE7 X-ray structure**. **a) **Intermolecular contact map of the Im7-ColE7 X-ray structure (PDB code: 7CEI) obtained by COCOMAPS [18], and **b) **consensus map of the 2000 MD snapshots. The ICs discussed in the text are outlined by an arrow and labeled.

*Structural overview*. The structure includes the C-terminal DNase domain of *E.coli *colicin E7 (ColE7), from residue 447 to 573, and the full length (residues 1-87) Im7 immunity protein. Colicins have cytotoxic activity against their target cells, which can be inhibited by their cognate immune proteins. The binding of colicins to their cognate immunity proteins are among the strongest recorded for protein-protein interactions and is also highly specific, being the cytotoxicity of each colicin only completely inhibited by its cognate immunity protein. Interestingly, colicins with altered specificity may potentially be used as antibacterial agents [27,28], and the ColE7-Im7 complex has indeed been object of extensive design studies [29,30]. The interacting surface of the ColE7-Im7 complex is highly charged, and also charge-complementary, while the surface complementarity is not so obvious [25]. Im7 has a V-shaped structure, extending two arms to clamp the ColE7 (see Figure 2). One arm (the one presenting the larger sequence variation among members of immunity proteins in the same subfamily) mainly uses acidic side-chains to interact with the basic side-chains of ColE7. The second arm is more conserved and interacts with the partner using also main-chain atoms. From the contact map of the X-ray structure, reported in Figure 3a, it is clear that Im7 and conE7 proteins interact through their central regions, i.e. residues 510-545 of ColE7 and 20-65 of Im7 (with residues 20-50 belonging to the first and residues 51-65 belonging to the second arm).

As already mentioned, the interaction between the two partners is very specific and strong, presenting an experimental Gibbs free-energy of binding equal to -19.50 kcal/mol [24]. Both proteins present high percentage of hydrophilic residues at the binding interface. Our COCOMAPS [18] analysis revealed that 35 of the total 52 ICs indeed occur between two hydrophilic residues, while only one of them involves the two hydrophobic residues significantly buried at the interface, namely Val27/Im7 and Val523/colE7. Importantly, fifteen direct hydrogen bonds are found at the interface, five of them being salt bridges. The Asn26 and Asp31 residues of Im7 contribute significantly to the H-bonds network, the former one participating in three H-bonds, the latter one giving two salt bridges with ColE7 Arg520 and Lys525. Clearly, the charged residues at the interface play an important role in the interaction. It was shown that the triple mutant of Im7 where Asp31, Asp35 and Glu39 are mutated to Asn/Gln completely lacks *in vivo *inhibitory activity against ColE7, with inhibitory effect of the residues in the order Asp31 > Asp35 > Glu39 [25]. Additionally, two Im7 tyrosines in the middle of the biological interface, Tyr55 and Tyr56, also seem to play a crucial role [25]. They contribute one intermolecular H-bond each, but also give hydrophobic interactions with ColE7. In particular, Tyr55 is 98% and Tyr56 73%buried upon the complex formation, being inserted into ColE7 non-polar pockets (Figure 2).

*MD simulations and MDcons analysis*. As an indicative measure of the stability and conformational drift of the complex in the simulation, the RMSD of the C-alpha atoms from their initial position was monitored as a function of the simulation time and is reported in the Additional file 1. The RMSD trend quickly reaches a plateau at 1.5 Å after the equilibration steps, indicating that the system remains stable during the 100-ns long simulation. The radius of gyration Rγ, a property linked to the molecular volume and compactness, is also very stable after few ns, showing a very limited variation and indicating that the complex does not undergo significant conformational changes (Additional file 1). The good convergence of the simulation is reflected by the high root mean square inner product (RMSIP) value, 0.79, calculated between the first 10 principal component vectors of the two halves of the trajectory.

The overall conservation of the interface during the dynamics can be visually appreciated by the comparison of the MD consensus map and the inter-molecular contact map of the X-ray structure (reported in Figure 3) and is reflected by the C_70 _and C_90 _values. The MDcons analysis results in C_70 _and C_90 _values of 0.84 and 0.69, which means that 84% and 69% of the ICs are conserved in at least 70% and 90% of the frames, respectively, thus indicating a limited flexibility of the interface during the dynamics. In particular, of the total 52 ICs present in the X-ray structure, 19 are maintained in all the examined frames (CR_kl _= 1, Additional file 2). These include contacts between residues involved in 9 out of the total 15 direct inter-molecular hydrogen bonds. The interactions between Tyr55/Im7 and the side chain of Lys528/ColE7, and between Pro57/Im7 and Ser514/ColE7 are also fully conserved. As a remark, these contacts are driven by van der Waals interactions, for example between the aliphatic part of the Lys528 side chain and the aromatic ring of Tyr55, with no evidence of a H-bond or a π-cation interaction between the amino group of Lys528 and the OH group or the aromatic ring of Ty55 in the examined frames. This highlights that tools based on the detection of standard interaction motifs, such as H-bond or π-cation interaction, would completely miss this strongly maintained contact [31].

The remaining 33 ICs exhibit CR_kl _in the range 0.99 to 0.07. The least conserved ICs, among those observed in the X-ray structure, correspond to Asn26/Im7-Val523/ColE7 (CR_kl _= 0.14), Leu34/Im7-Lys525/ColE7 (CR_kl _= 0.13), Asp35/Im7-Thr539/ColE7 (CR_kl _= 0.10) and Asp35/Im7-Lys528/ColE7 (CR_kl _= 0.07). The first three interactions are non-specific and located at the external boundaries of the binding interface. Contributing residues are at a minimum distance above 4 Å in the X-ray structure. Therefore their low conservation is consistent with a small fluctuation of the two proteins around their recognition interface. The last one is instead surprisingly a salt bridge, which is clearly completely lost during the MD simulations. As we contemporarily found a conservation rate of 0.90 between Asp35/Im7 and Lys537/ColE7 giving no H-bond in the X-ray structure (Additional file 2), we decided to monitor the distances between the functional groups of Asp35 of Im7and Lys528/Lys537 of ColE7 in the time, from which it is clear that the salt-bridge between Asp35/Im7 and Lys528/ColE7 is rapidly lost during the simulation and replaced by a Asp35/Im7 salt-bridge with Lys537/ColE7 (Additional file 1). Lys537/ColE7 also establishes a salt-bridge with Glu39/Im7 that is more than 5 Å apart in the X-ray structure (see below), as shown in the inset of Figure 2 for frame 113, corresponding to 5.65 ns of simulation. Therefore, quite surprisingly, some variability in the complex salt-bridge network emerges during the dynamics simulation, around the crucial negatively charged Asp35/Im7 residue.

**Figure 4:**
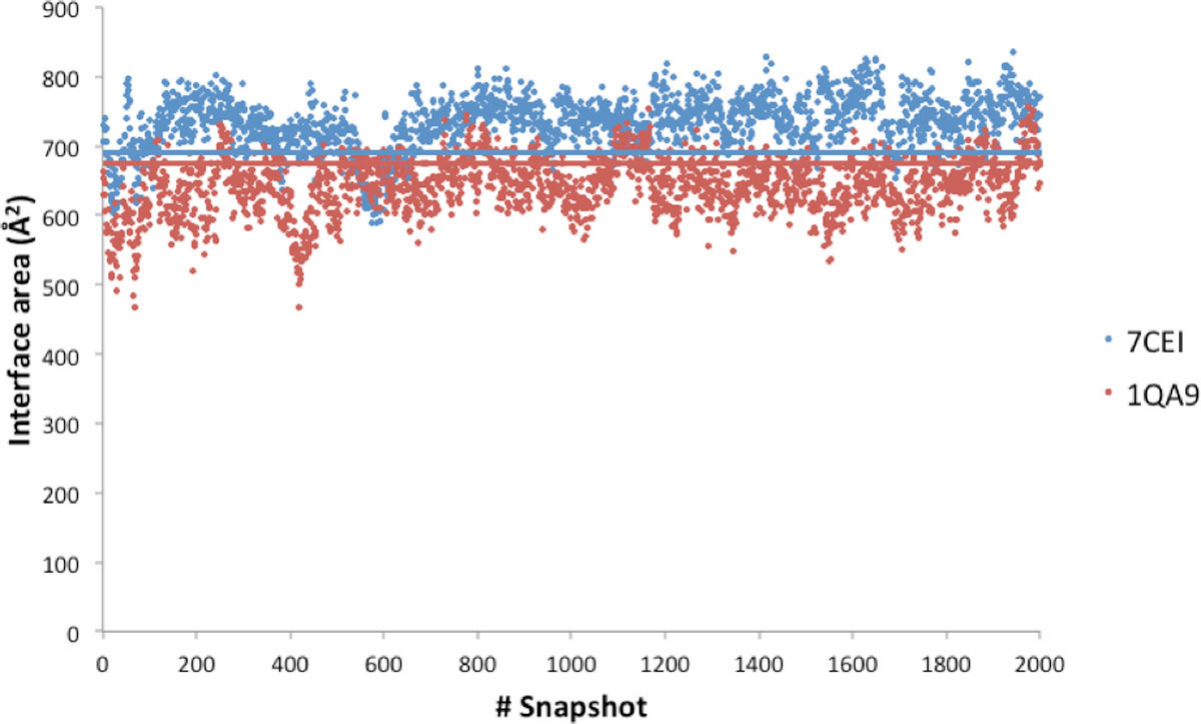
**Interface area of the two complexes along the MD simulations**. The two straight lines correspond to the interface area in the corresponding X-ray structures. Average values are reported in Table 1.

Interestingly, during the dynamics some additional ICs raise, which are not present in the starting X-ray structure (see Additional file 2). In total we observe 41 of these contacts, but the majority of them are observed only in a small fraction of snapshots, as only 6 of them have a conservation rate of 0.50 or above. Only two 'novel' interactions are very well conserved during the simulations, having a conservation rate around 0.8, they are Glu39/Im7-Lys537/ColE7 (see above) and Pro48/Im7-Thr531/ColE7. Such ICs are found at the margins of the interface area and are mostly located in flexible regions of the two molecules, however, as outlined above, one of them interestingly involves charge complementary residues. Overall, the interface area slightly expands during the dynamic simulations. The average interface area of the 2000 snapshots is 733 Å^2 ^(with a standard deviation of 38), which compares with the value of 692 Å^2 ^in the X-ray structure [18], see Figure 4.

### CD2-CD58 complex (1QA9)

**Figure 5:**
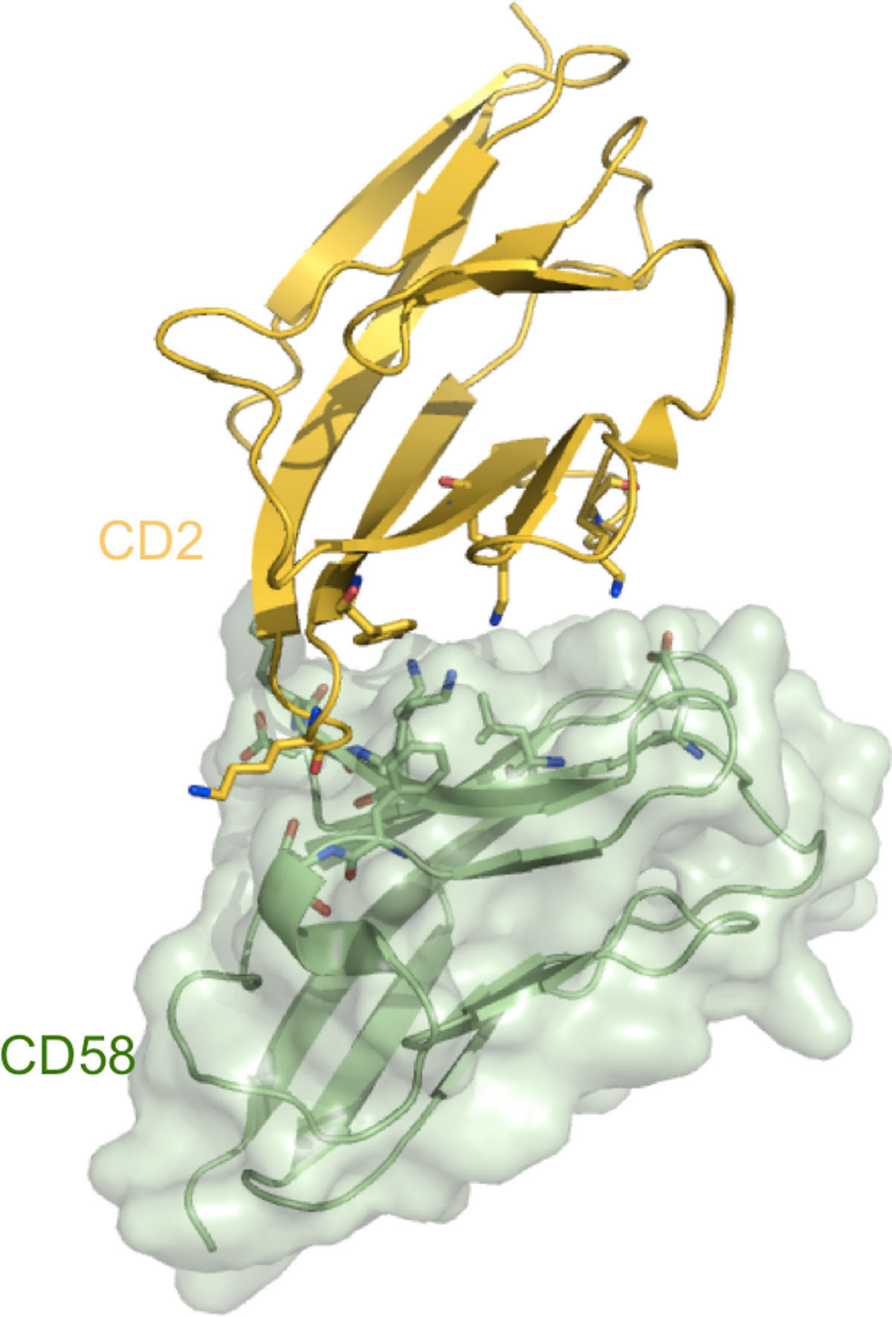
**A 3D representation of the CD2-CD58 complex (PDB ID: 1QA9)**. Residues involved in ICs with CR_kl _= 1 are shown in a stick representation.

*Structural Overview*. The second crystal structure we analyzed is the heterophilic adhesion complex between the amino-terminal domain of human CD2 (hCD2) and its counter-receptor CD58 [26]. CD2 is a transmembrane cell surface glycoprotein found on virtually all T cells, thymocytes and NK cells and promotes the initial stages of opposing cells contact, optimizing immune recognition. The structure includes the N-terminal adhesion domain of human CD2, composed of 102 residues (precisely residues 4-105), and the N-terminal adhesion domain of CD58 composed of 95 residues. It is worth noting that strategies aimed to block the CD2-CD58 interaction are being developed to treat autoimmune diseases [32-37], with a recombinant CD58-Ig fusion protein already approved for the treatment of chronic plaque psoriasis in adults [32,36]. As already mentioned, the binding affinity of the immunoglobulin (Ig)-like domains CD2-CD58 is relatively low, with an experimental Gibbs free-energy of binding equal to -7.16 kcal/mol [24,38-41]. Moreover, very rapid k_off _and k_on _rates have been measured for the system [42,43]. Actually, in this complex a strikingly asymmetric, orthogonal, face-to-face interaction is revealed, involving the major β-sheet of the respective immunoglobulin- like domains with poor shape complementarity (see Figure 5) [26].

**Figure 6:**
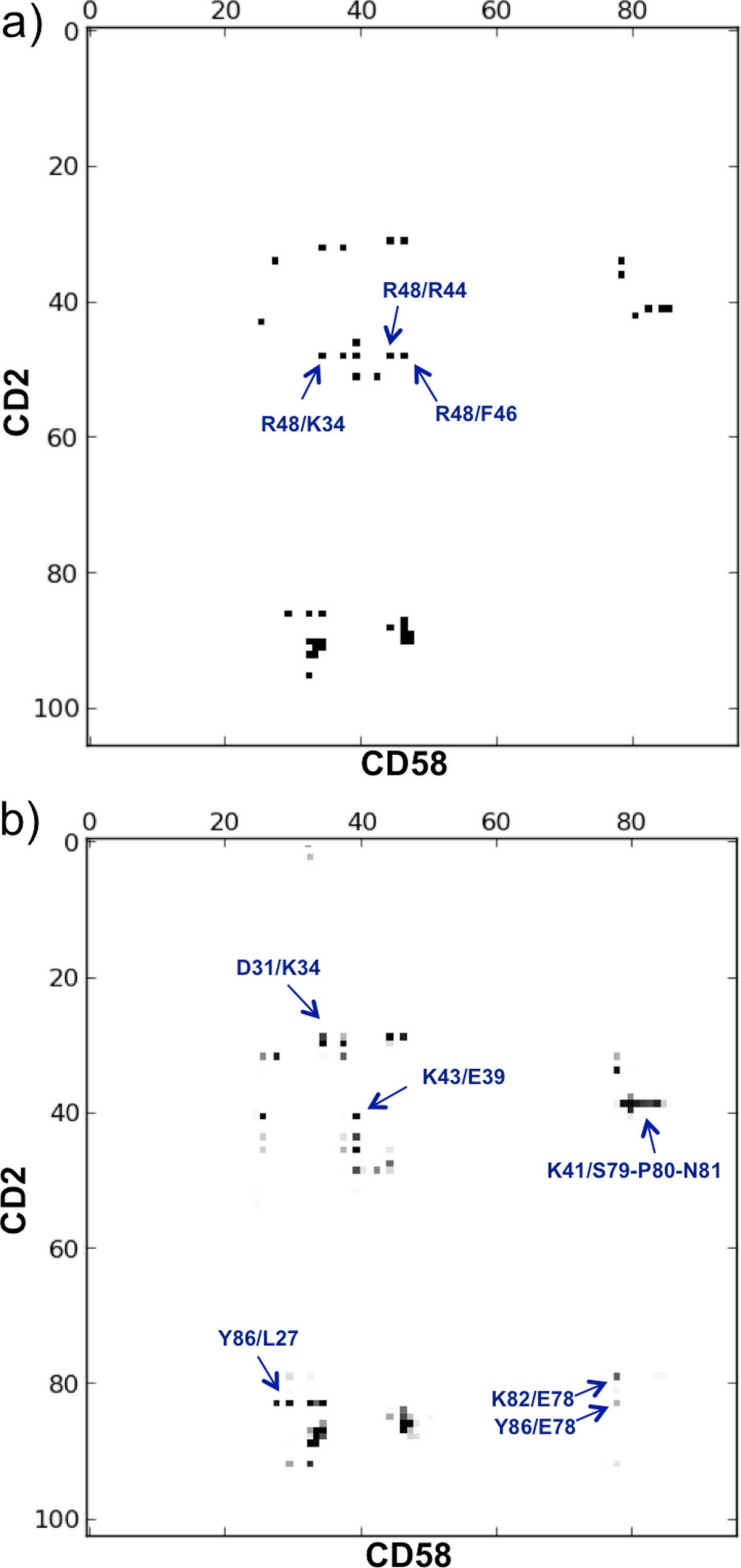
**Comparison between the MDcons consensus map and the intermolecular contact map of the CD2-CD58 X-ray structurea)** Intermolecular contact map of the CD2-CD58 X-ray structure (PDB code: 1QA9) obtained by COCOMAPS [18], and **b)** consensus map of the 2000 MD snapshots. The ICs discussed in the text are outlined by an arrow and labeled.

From the contact map of the X-ray structure (Figure 6a), we can see at a glance that CD58 mainly uses its 20-50 region for the interaction, plus a small segment around residues 78-85, whereas CD2 interacts with regions 25-50 and 80-100. As reported in the X-ray structure reference [26], the CD2-CD58 complex presents fifteen hydrogen bonds at the interface, including 10 salt-bridges, involving the CD2 residues Arg48, Lys51 and Asp31 and the CD58 residues Glu37, Glu39 and Arg44. Only three hydrophobic residues are found at the interface, Phe46/CD58, Pro80/CD58 and Tyr86/CD2, each involved in few van der Waals contacts. The interface is thus dominated by charged and hydrophilic residues. In fact, by COCOMAPS analysis, we found that 25 of the total 38 ICs characterizing the complex occur between two hydrophilic residues, corresponding to the 66% of them, while only one of them involves two hydrophobic residues. The interface area of the X-ray structure is 676 Å^2^.

*MD simulations and MDcons analysis*. RMSD of the C-alpha atoms from their initial position, and the radius of gyration Rγ of the system are reported in the Additional file 1. Both measures indicate that the complex is stable during the dynamics simulation. The RMSD values are pretty stable on values between 1.5 and 2.0 Å, although more fluctuating than the 7CEI ones in the first half of the simulation, where it reaches maximum values of 2.5 Å. The radius of gyration Rγ is also stable. It assumes an absolute value very close to that of the 7CEI system, although showing a slightly higher fluctuation. Also for this simulation, a high RMSIP value, 0.80, was calculated, indicating that it is very well converged.

The C_70 _and C_90 _parameters assume the values of 0.78 and 0.49, respectively (Table 1), that means that only 78% of ICs are maintained in at least 70% of the frames and only 49% in at least 90% of them, indicating a certain variability of the ICs during the simulation. This is also apparent from the complex consensus map (Figure 6b), where it is clear that additional spots appear as compared to the contact map of the X-ray structure, i.e. additional ICs are established during the simulation, while most ICs of the X-ray structure are not fully conserved (appear as gray spots in the consensus map).

Of the 38 ICs observed in the X-ray structure, only 9 (24%) are indeed rigidly maintained (CR_kl _= 1) during the MD simulation. These include only 4 of the 15 intermolecular hydrogen bonds, showing that the remaining 11 hydrogen bonds cannot be established at least in some of the explored frames, as the corresponding residues are more that 5 Å apart. Among the 100% conserved ICs there are also some substantially hydrophobic ones, which cannot be easily characterized with standard analysis tools based on, for example, the characterization of hydrogen-bonds at the interface. For instance, the ICs between Phe46/CD58 and Gly90/CD2 and the one between the aromatic ring of Tyr86/CD2 and the aliphatic portion of Lys34/CD58 have a CR_kl _= 1.0. The remaining 31 ICs of the starting X-ray structure exhibit CR_kl _in the range 0.99 to 0.00. The least conserved ICs, among those observed in the X-ray structure, Arg48/CD2-Arg44/CD58 (CR_kl _= 0.11), Arg48/CD2-Phe46/CD58 (CR_kl _= 0.00) and Arg48/CD2-Lys34/CD58 (CR_kl _= 0.00), all involve Arg48 of CD2 in the central region of the CD58 binding interface (see Additional file 2). The disruption of this network of interactions is probably due to repulsive electrostatic interaction between Arg47/CD2 on one side, and Arg44 and Lys34 of CD58 on the other side.

Interestingly, during the dynamics 59 additional ICs are established, which are not present in the starting X-ray structure, as can easily be seen from the corresponding consensus map shown in Figure 6b. Ten of them have a CR_kl _above 0.50 and 5 of them are very well conserved, exhibiting CR_kl _values above 0.80. They include ICs made by residues located at the boundary of the binding interface, such as Lys41/CD2 and Lys43/CD2, andPro80/CD58 and Asn81/CD58, but also residues in the middle of the interface, such as Tyr86 in CD2 and Leu27 and Glu39 in CD58 (Additional file 2). From the consensus map (Figure 6b), it is also easy to spot a new contact region around residue 80 of both proteins. In particular, novel contacts emerge between Lys82/CD2 and Glu78/CD58 (CR_kl _= 0.63) and between Tyr86/CD2 and Glu78/CD58 (CR_kl _= 0.29). These also are ICs in the middle of the binding interface.

In a small fraction of snapshots (1-3%), we observed ICs between residues, such as Lys91/CD2 and Phe49/CD58, which are more than 10 Å apart in the crystallographic structure, clearly indicating a significant flexibility of the complex interface, not captured by the RMSD or the Rγ. Differently from 7CEI, for 1QA9 the interface area is slightly reduced during the dynamics. The average interface area of the 2000 snapshots is indeed 641Å^2 ^(with a standard deviation of 41), that is 35 Å^2 ^less than the X-ray one, 676Å^2 ^[18], see Figure 4.

### Comparison between the two complexes

MDcons analysis of the two structures highlights a higher flexibility and variability in the interface of CD2-CD58 relative to that of Im7-ColE7. This can easily be appreciated by comparing the consensus maps obtained for the two systems (and by their comparison with the inter-molecular contact maps of the corresponding X-ray structures, see Figures 3,6) and is reflected in the lower values of C_50_, C_70 _and C_90 _parameters obtained for CD2-CD58 (Table 1). Specifically, while the C_50 _and C_70 _of CD2-CD58 are marginally smaller than those calculated for Im7-ColE7, its C_90 _is significantly smaller (0.49 *vs*. 0.69). Furthermore, only 9 ICs at the interface of the CD2-CD58 complex, corresponding to 24% of the contacts observed in the X-ray structure, are 100% conserved during the dynamics, which compares with the 19 fully conserved ICs at the interface of Im7-ColE7, corresponding to 37% of the contacts of the X-ray structure (Tables S1, S3). Finally, the fraction of X-ray contacts with a CR_kl_< 0.50 is 12% for 7CEI, indicating that only a few contacts are basically lost during the MD simulation, a value that increases to the clearly higher value of 26% for 1QA9.

Overall, the above analysis clearly indicates that the ICs of 1QA9 are somewhat more flexible than those of 7CEI, highlighting a more dynamic behavior of the interface of 1QA9. Furthermore, the average interface area of 1QA9 is reduced relative to the X-ray structure during the MD simulation, whereas the 7CEI one experiences a small increase (Figure 4).

Both systems acquire novel ICs during the MD simulations, not observed in the X-ray structure (Tables S2,S4). Precisely, 59 and 42 novel contacts are observed for 1QA9 and 7CEI, respectively. However, in CD2-CD58 the novel established ICs do not compensate the ones lost compared to the starting X-ray structure and the interface area is consequently reduced by about 35 Å^2^. On the contrary, the interface area of the Im7- ColE7 complex is increased on average by 40 Å^2 ^during the MD simulation.

Once they were outlined by MDcons, we could follow and characterize the ICs that exhibited a significant variation compared to the X-ray structure by classical analysis tools, such as distance monitoring along the simulation. Results of this analysis surprisingly showed that the salt-bridge network of Im7-ColE7 is quite dynamic, with Asp35/Im7 losing its H-bond with Lys528/ColE7 during the dynamics, and acquiring one novel H-bond with Lys537/ColE7, which in turns rearranges its side-chain to also give a H-bond with Glu39/Im7.

It is interesting that we found a poor conservation in terms of ICs for the interface of the human CD2-CD58 complex and a decrease in its extension, compared to the static 3D structure, as it has been experimentally shown to have very rapid on and off kinetic rates and to exchange to new partners, after dissociation, rather than rebind to the same CD2-CD58 pair components [39]. The rapid off rate of CD2-CD58 has previously been hypothesized to be related to the virtual absence of hydrophobic contacts at the interface. However, this is a feature that such complex shares with many other complexes, including the Im7-ColE7 one analysed here. We suggest that the CD2-CD58 peculiar binding behavior could also be related to the dynamics of the CD2 and CD58 proteins in the complex, that spontaneously lose a significant fraction of contacts while exploring novel ones.

## Conclusions

We performed MD simulations on two protein complexes with interfaces of comparable area and both dominated by hydrophilic residues. To analyse the results of the simulations we used some classical tools and a novel tool, MDcons (freely available for download at https://www.molnac.unisa.it/BioTools/mdcons/index.php/MDcons), we specifically developed for the analysis of the MD trajectories of protein complexes. The two systems under analysis correspond to the high binding affinity Im7-ColE7 structure and to the low affinity CD2-CD58 complex. Analysis through the MDcons tool allowed us to outline interesting dynamic features at the interface of both the analysed complexes, evidencing overall a more rigid interface for Im7-ColE7, and a relatively more flexible and dynamic interface for the CD2-CD58 complex. We have thus demonstrated the utility of MDcons in complementing classical analysis tools, when studying the dynamics of protein complexes. This approach can also be straightforwardly applied to other macromolecular complexes, such as DNA-protein and RNA-protein complexes.

## Methods

### MD simulations

The starting coordinates of the Molecular Dynamics (MD) simulations of the two protein complexes selected for this study were the PDB codes: 7CEI and 1QA9 (resolution 2.30 and 3.20 Å^2^, respectively). MD simulations were performed using the GROMACS 4.6.2 package with the Amber99SB-ILDN force field [44]. The complexes were slightly relaxed using 50 steps of steepest descent minimizer. The systems were then immersed in an explicit TIP3P water cubic box [45,46], under periodic boundary conditions, which extended at least 10 Å away in each direction from any atom of the complex and contained 14267 and 12074 water molecules for the 7CEI and 1QA9 systems, respectively. Four sodium ions were added to the 1QA9 system and three chloride ions to the 7CEI system, to neutralize them as needed for the particle mesh Ewald calculation [47] of the long-range electrostatic interactions, while a cut-off of 10 Å was used for van der Waals and short-range electrostatic interactions. 500 steps of steepest descent minimization were performed to remove bad contacts with the solvent. All bonds involving hydrogen atoms were constrained by the LINCS algorithm [48]. Equilibration of the solvent and ions around the complexes with position constraints of the heavy atoms was performed for 1 ns at constant temperature (300 K) in the NVT ensemble, followed by 1 ns at constant temperature (300 K) and pressure (1 atm) in the NPT ensemble. NVT simulations were carried out using the velocity rescaling thermostat (V-rescale) [49] and the NPT ones using the Parrinello-Rahman barostat [50]. After equilibration, 100 ns MD simulations were performed in the NPT ensemble. Root mean square deviation from the X-ray coordinates (RMSD), the root mean square fluctuation (RMSF) and the radius of gyration were calculated with standard GROMACS tools. To assess the convergence of the trajectories, we split the trajectories into two halves, and we calculated the root mean square inner product (RMSIP) between the first 10 eigenvectors of PCA analysis on the two halves [51,52]. The calculated RMSIP values are 0.79 for the 7CEI and 0.80 for the 1QA9 trajectory. 2000 snapshots were then generated for each system from the whole trajectory of 100 ns, by writing the coordinates every 50 ps, for further MDcons analyses. For comparison purposes, one more independent 100 ns-long NPT simulation was run for each system with different initial velocities. For each system, the similarity between the two trajectories was assessed in terms of RMSIP on the first 10 eigenvectors from PCA analysis. The obtained RMSIP value was 0.76 for 7CEI and 0.74 for 1QA9. RMSD and gyration radius values along the simulations were also compared for each system (Fig. S3). As the two trajectories for each system resulted to be very similar, for the sake of simplicity the analysis reported in the text was limited to the first trajectory for each system.

### MDcons

The MDcons package consists of a collection of fortran sources and python scripts. It is freely available for download at the wiki page: https://www.molnac.unisa.it/BioTools/mdcons/index.php/MDcons. It takes as input a set of PDB snapshot files or a single PDB trajectory file representing the protein-protein complex conformations during the MD simulations. MDcons then gives as output: i) a consensus map which represents the frequency of inter-residue contacts for all the MD simulation; ii) a list of the inter-residue contacts with the relative conservation rate during the MD simulations; iii) the C_50_, C_70 _and C_90 _coefficients, which represent respectively the number of inter-residue contacts conserved for at least 50, 70 and 90% of the analysed frames.

In the analysis, two residues are considered in contact if at least two heavy atoms are at a distance shorter than 5 Å. This is the definition used in the CAPRI experiment, and we used it for the sake of consistency with a well established and largely accepted protocol [5,6,22]. The conservation rate for each inter-residue pair is defined in Eq. 1:

(1) $$CR_{kl}=nc_{kl}/N,$$

Where *nc*_kl _is the total number of frames where residues *k *of protein A and *l *of protein B are in contact, and N is the total number of analysed frames. The conservation rate thus ranges between CR_kl _= 0, if the contact between residues *k *and *l *never happens, to CR_kl _= 1, if the contact is present in all the frames.

C_70 _is calculated as in Eq. 2, where *nc*_70 _is the total number of inter-residue contacts conserved in at least 70 % of the analysed frames and *nc*_j _is the total number of inter- residue contacts in frame *i*. C_50 _and C_90 _are calculated similarly.

(2) $$C_{70}=\frac{nc_{70}}{\overset{N}{\underset{i}{\sum }}\frac{nc_{i}}{N}}$$

Finally, the interface area for each snapshot is estimated as half of the Accessible Surface Area (ASA) buried upon complex formation as calculated by NACCESS [53].

## Competing interests

The authors declare that they have no competing interests.

## Authors' contributions

SA carried out the MD simulations and carried out the analyses. EC wrote the code and carried out the analyses. AV wrote the code, carried out the analyses and helped to draft the manuscript. RO and LC conceived of the study, participated in its design and coordination and drafted the manuscript. All authors read and approved the final manuscript.

## Supplementary Material

Additional file 1 **Classical molecular dynamics analyses**. Figures reporting the RMSD and gyration radius during the 100-ns MD simulations for the 7CEI and 1QA9 systems and the distance between specific H-bond donor and acceptor atoms at the interface of the 7CEI system. Click here for file

Additional file 2 **Inter-residue contacts with relative conservation rates**. Tables reporting the conservation rates, CR_kl_, of the 7CEI and 1QA9 inter-residue contacts. Click here for file
