# Supplementary material for: MDcons: Intermolecular contact maps as a tool to analyze the interface of protein complexes from molecular dynamics trajectories
Source: BMC Bioinformatics. 2014 May 6;15(Suppl 5):S1. doi: 10.1186/1471-2105-15-S5-S1 (PMC4095001; doi:10.1186/1471-2105-15-S5-S1)
Supplement: Additional file 1 — Classical molecular dynamics analyses. Figures reporting the RMSD and gyration radius during the 100-ns MD simulations for the 7CEI and 1QA9 systems and the distance between specific H-bond donor and acceptor atoms at the interface of the 7CEI system. [file 1471-2105-15-S5-S1-S1.pdf]

Additional file 1 for "*MDcons: Intermolecular contact maps as a tool to analyze the interface of protein complexes from molecular dynamics trajectories*" by Abdel-Azeim S. *et al*.

Table S1. List of ICs observed in the X-ray structure of Im7-ColE7 (7CEI), with relative conservation rates, CR_{kl}, in the 2000 MD snapshots.

	Im7			ColE7		X-ray Dist	CRkl
LEU	34	A	LYS	525	B	4.99	1.00
VAL	27	A	VAL	523	B	3.82	1.00
VAL	27	A	ASP	519	B	3.66	1.00
VAL	27	A	ARG	520	B	4.77	1.00
VAL	27	A	LYS	525	B	4.30	1.00
TYR	56	A	PHE	541	B	3.66	1.00
TYR	56	A	LYS	528	B	2.72	1.00
TYR	56	A	ASN	517	B	3.43	1.00
TYR	56	A	SER	514	B	4.24	1.00
TYR	56	A	THR	529	B	3.26	1.00
TYR	56	A	ARG	530	B	3.47	1.00
TYR	55	A	SER	514	B	2.36	1.00
TYR	55	A	ASN	517	B	3.82	1.00
TYR	55	A	LYS	528	B	3.81	1.00
TYR	55	A	ARG	520	B	3.81	1.00
TYR	55	A	ASN	516	B	3.70	1.00
THR	30	A	LYS	525	B	3.24	1.00
PRO	57	A	SER	514	B	3.67	1.00
LEU	38	A	LYS	528	B	3.99	1.00
LEU	34	A	LYS	528	B	3.92	0.99
LEU	34	A	ARG	520	B	3.39	0.99
ILE	54	A	ASN	516	B	3.61	0.99
GLU	23	A	ASN	516	B	3.21	0.99
ASP	63	A	ARG	515	B	2.77	0.98
ASP	52	A	ARG	530	B	3.28	0.96
ASP	52	A	THR	531	B	2.91	0.96
ASP	49	A	THR	531	B	3.19	0.96
ASP	31	A	ARG	520	B	2.84	0.95
ASP	31	A	LYS	525	B	2.65	0.94
ASN	26	A	ARG	520	B	3.29	0.94
ASN	26	A	LYS	525	B	3.11	0.93
ASN	26	A	ASP	519	B	4.28	0.92
ASN	26	A	ASN	517	B	4.66	0.91
ASN	26	A	ASN	516	B	2.75	0.90
ALA	29	A	LYS	525	B	4.21	0.89
ALA	28	A	LYS	525	B	2.52	0.88
THR	51	A	THR	531	B	3.43	0.78
ASP	52	A	THR	529	B	4.30	0.77

TYR	56	A	PHE	513	B	4.88	0.73
ASP	63	A	SER	514	B	3.70	0.69
ASP	35	A	LYS	537	B	4.27	0.66
ASP	31	A	LYS	528	B	3.93	0.65
ILE	54	A	SER	514	B	4.22	0.64
GLU	25	A	LYS	525	B	3.74	0.62
ASP	63	A	ASN	516	B	4.61	0.58
GLU	23	A	ASP	519	B	4.59	0.53
ASP	35	A	LYS	528	B	2.91	0.39
ASP	31	A	SER	540	B	4.35	0.17
GLY	50	A	THR	531	B	4.28	0.14
ASN	26	A	VAL	523	B	4.69	0.13
ASP	35	A	THR	539	B	4.26	0.10
TYR	56	A	ASN	466	B	4.96	0.07

Table S2. List of ICs not observed in the X-ray structure of Im7-ColE7 (7CEI), with relative conservation rates, CR_{kl}, in the 2000 MD snapshots.

	Im7			ColE7		CRkl
PRO	48	A	THR	531	B	0.80
GLU	39	A	LYS	537	B	0.77
ASP	52	A	GLN	532	B	0.64
GLU	23	A	ARG	515	B	0.64
SER	64	A	ARG	515	B	0.61
PRO	65	A	ARG	515	B	0.48
ASN	26	A	LYS	528	B	0.40
ASP	49	A	GLN	532	B	0.24
TYR	56	A	PRO	527	B	0.23
THR	51	A	THR	529	B	0.19
ASP	62	A	ARG	515	B	0.14
ALA	28	A	VAL	523	B	0.13
TYR	55	A	THR	529	B	0.10
ILE	68	A	ASP	516	B	0.10
ASP	31	A	THR	539	B	0.08
ASP	63	A	PHE	513	B	0.07
LYS	20	A	ARG	515	B	0.06
ASP	32	A	LYS	537	B	0.06
GLU	25	A	ARG	520	B	0.06
ASP	49	A	ARG	530	B	0.05
TYR	56	A	GLN	512	B	0.04
VAL	27	A	LYS	522	B	0.04
ILE	22	A	ASP	516	B	0.03
PRO	57	A	ARG	530	B	0.02
THR	51	A	LYS	528	B	0.02
ALA	28	A	ARG	520	B	0.02
ALA	29	A	LYS	497	B	0.01
TYR	56	A	LEU	465	B	0.01
PRO	57	A	ASP	516	B	0.01

LEU	19	A	ASP	516	B	0.01
ILE	54	A	ASN	517	B	0.01
ASP	31	A	ALA	526	B	0.01
ASP	35	A	GLY	536	B	0.01
THR	51	A	VAL	534	B	0.01
PRO	48	A	GLN	532	B	0.01
ALA	29	A	VAL	523	B	0.01
LEU	38	A	THR	539	B	0.01
VAL	36	A	LYS	537	B	0.01
GLU	39	A	GLY	536	B	0.01
ASP	63	A	ASN	517	B	0.01
TYR	56	A	LYS	463	B	0.01

Table S3. List of ICs observed in the X-ray structure of CD2-CD58 (1QA9), with relative conservation rates, CR_{kl}, in the 2000 MD snapshots.

	CD2			CD58		X-ray Dist	CRkl
TYR	86	A	LYS	29	B	2.83	1.00
TYR	86	A	LYS	34	B	3.85	1.00
TYR	86	A	LYS	32	B	3.62	1.00
LYS	91	A	LYS	34	B	4.90	1.00
LYS	91	A	ASP	33	B	3.44	1.00
LYS	89	A	SER	47	B	2.73	1.00
LYS	89	A	PHE	46	B	3.43	1.00
LYS	43	A	GLU	25	B	3.25	1.00
LYS	34	A	LEU	27	B	3.76	1.00
GLY	90	A	LYS	34	B	2.66	0.99
GLY	90	A	ASP	33	B	3.20	0.99
GLY	90	A	PHE	46	B	3.31	0.98
ASP	32	A	LYS	34	B	3.23	0.98
ASP	31	A	PHE	46	B	4.67	0.97
ASN	92	A	LYS	32	B	2.70	0.95
ASN	92	A	ASP	33	B	3.04	0.93
ARG	48	A	GLU	39	B	3.53	0.93
ASP	32	A	GLU	37	B	4.89	0.87
ASP	31	A	ARG	44	B	3.13	0.86
LYS	42	A	PRO	80	B	3.10	0.86
LYS	41	A	ASP	84	B	3.34	0.82
LYS	51	A	GLU	39	B	2.62	0.81
GLU	36	A	GLU	78	B	4.35	0.77
GLU	95	A	LYS	32	B	3.25	0.74
THR	88	A	ARG	44	B	4.16	0.70
LYS	41	A	ILE	82	B	2.88	0.69
THR	88	A	PHE	46	B	4.46	0.67
ASP	87	A	PHE	46	B	4.90	0.56
LYS	34	A	GLU	78	B	4.02	0.46
GLY	90	A	SER	47	B	4.97	0.40
LYS	51	A	GLU	42	B	2.54	0.35

ARG	48	A	GLU	37	B	2.66	0.31
ARG	48	A	ARG	44	B	4.21	0.30
GLN	46	A	GLU	39	B	4.63	0.25
LYS	41	A	SER	85	B	2.95	0.19
ARG	48	A	LYS	34	B	2.30	0.11
GLY	90	A	LYS	32	B	4.99	0.00
ARG	48	A	PHE	46	B	3.68	0.00

Table S4. List of ICs <u>not</u> observed in the X-ray structure of CD2-CD58 (1QA9), with relative conservation rates, CR_{kl}, in the 2000 MD snapshots.

	CD2			CD58		CRkl
LYS	41	A	PRO	80	B	0.96
LYS	43	A	GLU	39	B	0.95
LYS	41	A	SER	79	B	0.90
TYR	86	A	LEU	27	B	0.89
LYS	41	A	ASN	81	B	0.87
ASP	31	A	LYS	34	B	0.75
LYS	82	A	GLU	78	B	0.63
LYS	34	A	GLU	37	B	0.62
TYR	86	A	ASP	33	B	0.59
GLU	50	A	ARG	44	B	0.58
LYS	34	A	GLU	25	B	0.47
ASP	40	A	PRO	80	B	0.45
LYS	89	A	LYS	34	B	0.40
GLU	95	A	LYS	29	B	0.35
TYR	86	A	GLU	78	B	0.29
ASP	31	A	GLU	37	B	0.28
THR	4	A	LYS	32	B	0.26
THR	88	A	SER	47	B	0.25
ARG	48	A	GLU	25	B	0.19
GLN	46	A	GLU	25	B	0.19
LYS	91	A	SER	47	B	0.17
LYS	51	A	ARG	44	B	0.16
LYS	82	A	LYS	29	B	0.13
ASP	32	A	ARG	44	B	0.13
LYS	91	A	SER	48	B	0.13
GLN	46	A	GLU	37	B	0.13
GLU	95	A	GLU	78	B	0.10
LYS	91	A	LYS	32	B	0.10
LYS	89	A	SER	48	B	0.10
LYS	51	A	ASN	40	B	0.09
LYS	43	A	PRO	80	B	0.08
LYS	41	A	GLU	78	B	0.06
LYS	82	A	LYS	32	B	0.05
SER	84	A	GLU	78	B	0.04
LYS	82	A	SER	85	B	0.04
THR	88	A	LYS	50	B	0.03
LYS	43	A	LYS	24	B	0.03
LYS	34	A	LYS	34	B	0.03

LYS	82	A	ASP	84	B	0.03
LYS	42	A	LYS	24	B	0.02
GLU	56	A	LYS	24	B	0.02
ASP	87	A	ARG	44	B	0.02
PHE	54	A	GLU	39	B	0.02
SER	84	A	LYS	29	B	0.02
GLU	50	A	GLU	42	B	0.01
LYS	43	A	GLU	37	B	0.01
ASN	92	A	LYS	34	B	0.01
ASN	5	A	LYS	32	B	0.01
ASN	92	A	LYS	29	B	0.01
LYS	91	A	PHE	49	B	0.01
LYS	42	A	GLU	78	B	0.01
GLU	95	A	GLU	76	B	0.01
SER	84	A	LYS	32	B	0.01
GLU	36	A	GLU	25	B	0.01
GLU	36	A	PRO	80	B	0.01
PHE	54	A	LYS	24	B	0.01
LYS	51	A	GLU	37	B	0.01
LYS	91	A	LYS	30	B	0.01
LYS	42	A	ASN	81	B	0.01
