# Supplementary material for: MDcons: Intermolecular contact maps as a tool to analyze the interface of protein complexes from molecular dynamics trajectories
Source: BMC Bioinformatics. 2014 May 6;15(Suppl 5):S1. doi: 10.1186/1471-2105-15-S5-S1 (PMC4095001; doi:10.1186/1471-2105-15-S5-S1)

Additional file 2 for "*MDcons: Intermolecular contact maps as a tool to analyze the interface of protein complexes from molecular dynamics trajectories*" by Abdel-Azeim S. *et al*.

Figure S1. Time dependence in the 100-ns MD simulations of RMSD for the Cα (on the left) and of gyration radius (Rg, on the right) for all the heavy atoms (on the right) for the two systems.

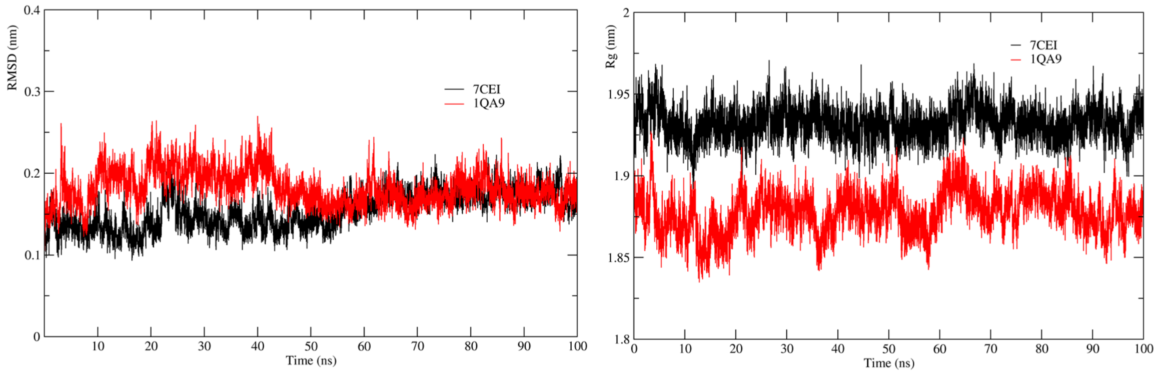

Figure S2. Time dependence of distances between donor atoms of the Asp35/im7 and Glu39/Im7 side-chains and acceptor atoms of the Lys528/ColE7 and Lys537/ColE7 side-chains.

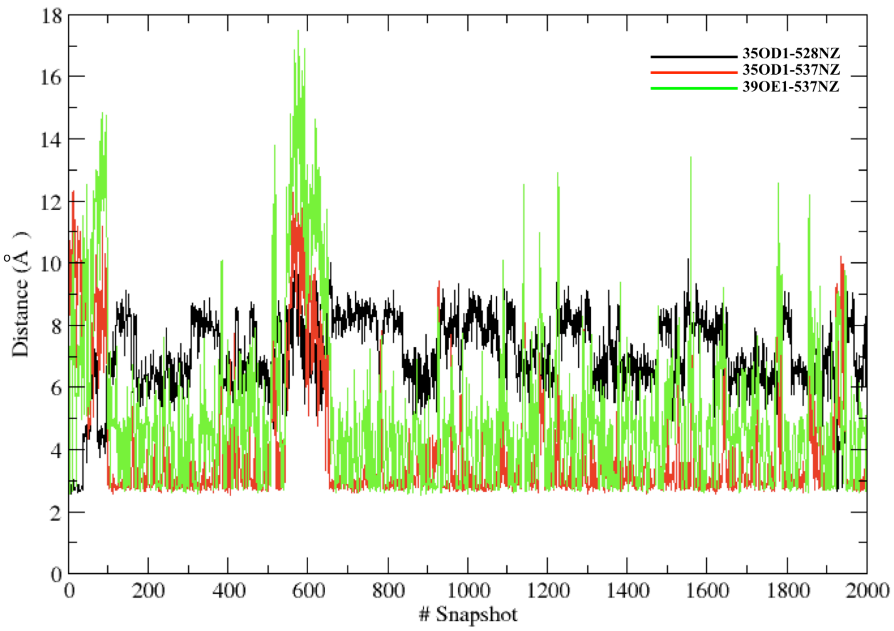

Figure S3. Comparison of the time dependence of RMSD for the Cα (on the left) and of gyration radius (Rg, on the right) for all the heavy atoms (on the right) between the two 100-ns MD simulations obtained for each of the two systems.

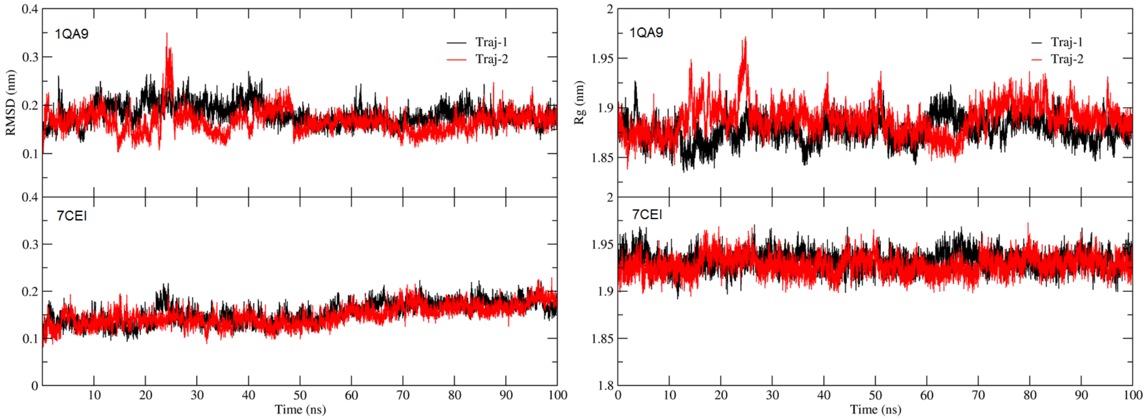

Supplement: Additional file 2 — Inter-residue contacts with relative conservation rates. Tables reporting the conservation rates, CRkl, of the 7CEI and 1QA9 inter-residue contacts. [file 1471-2105-15-S5-S1-S2.pdf]
